# The Impact of Oral Health and Dental Services on the Prevalence of Subjective Cognitive Decline Among Middle-Aged and Older US Adults: Behavioral Risk Factor Surveillance System, 2022

**DOI:** 10.5888/pcd22.250083

**Published:** 2025-09-04

**Authors:** Mohammed H Alshanbari, Amanda M Cheney, Hesham A Alhazmi, Erin D Bouldin

**Affiliations:** 1 Department of Health Management and Medical Informatics, Health Sciences College at Al-Leith, Umm Al-Qura University, Makkah, Saudi Arabia; 2 Division of Epidemiology, Department of Internal Medicine, University of Utah, Salt Lake City; 3 Informatics, Decision-Enhancement and Analytic Sciences Center, Department of Veterans Affairs Medical Center, Salt Lake City, Utah; 4 Department of Preventive Dentistry, Faculty of Dentistry, Division of Pediatric Dentistry, Umm Al-Qura University, Makkah, Saudi Arabia

## Abstract

**Introduction:**

Subjective cognitive decline (SCD) may be associated with poor oral health because of difficulty with self-care or comorbid conditions. Our study aimed to examine oral health status, use of dental services, and the prevalence of SCD among US middle-aged (45–64 y) and older (≥65 y) adults.

**Methods:**

We conducted a cross-sectional analysis of 2022 Behavioral Risk Factor Surveillance System (BRFSS) data. Our sample consisted of 83,479 adults aged 45 years or older who completed the cognitive decline module. The associations between SCD and oral health, use of dental services, sociodemographic characteristics, chronic disease conditions, and dementia risk factors were examined by using multivariate regression with a generalized linear model, survey-weighted to account for BRFSS’s complex sampling design. All models were stratified by age group (45–64 y vs ≥65 y).

**Results:**

Middle-aged and older adults with poor oral health had a higher weighted prevalence of SCD (13.6%) compared with those with good oral health (7.7%). After controlling for covariates, SCD prevalence was increased among adults aged 45 to 64 years with more tooth loss and was lower for those in the same age group who had visited a dentist in the past year (PR = 0.77; 0.65–0.90). Among people aged 65 years or older, SCD prevalence was significantly higher for people with tooth loss compared with no tooth loss, though this pattern was not linear.

**Conclusion:**

A significant association was found between the number of teeth lost, dental service use, and SCD, particularly among adults aged 45 to 64 years. Maintaining good oral health and having regular dental visits may be a strategy to reduce the risk of SCD in middle age. People should be encouraged to seek regular dental care.

**Summary**

**What is already known on this topic?**

Addressing modifiable risk factors may reduce dementia risk. Poor oral health, particularly periodontal disease and tooth loss, has been linked to subjective cognitive decline and Alzheimer disease, as a potential risk factor.

**What is added by this report?**

Data from the 2022 Behavioral Risk Factor Surveillance System show that middle-aged and older adults with poor oral health and those who did not use regular dental health services had a higher prevalence of subjective cognitive decline.

**What are the implications for public health practice?**

Health care professionals can emphasize the need for oral health care for middle-aged and older adults. Community-based dental health promotion programs may improve cognitive health and other health issues.

## Introduction

Subjective cognitive decline (SCD) is defined as a self-reported perception of worsening or more frequent episodes of confusion or memory loss over the past 12 months, independent of any cognitive clinician’s diagnosis or neuropsychological testing (1,2). SCD is increasingly recognized as an early sign of cognitive impairment, including Alzheimer disease and related disorders, and may precede the clinical diagnosis of more serious disorders (1–3). Not everyone with SCD will go on to develop Alzheimer disease or related disorders though a significant number eventually do (2,4,5).

Subjective cognitive decline is an important public health issue. Between 1996 and 2014, the prevalence of cognitive impairment among adults aged 50 years and older increased annually by an average of 0.7% in women and 1.0% in men (6). Taylor et al found 10.8% of people aged 45 to 64 years and 11.7% of those aged 65 years or older had SCD (2). Mild cognitive impairment prevalence is estimated to be 22% in community-dwelling adults aged 71 years or older (7,8). In 2019, an estimated 57 million people worldwide were living with dementia, and this number is projected to reach 153 million by 2050, emphasizing the urgent need for effective preventive methods (9). Therefore, it is imperative to identify SCD and its risk factors to enable effective interventions and timely prevention of mild cognitive impairment and its progression to dementia. Furthermore, early detection can also reduce medical and socioeconomic costs (8,10).

Another important global public health issue is tooth loss due to decay and gum disease in older adults (3) who tend to have poorer oral health than younger age groups, including a higher number of teeth lost, dental decay, and higher rates of gum and periodontal diseases (11,12). Additionally, challenges to accessing oral health care may contribute to this condition. Gupta et al found that 25% of adults had untreated tooth decay due to both financial and nonfinancial challenges (13). Many studies have shown significant associations between many systemic conditions and the oral cavity, including diabetes, arthritis, cardiovascular diseases, cirrhosis, and atherosclerosis (14,15). Additionally, researchers are increasingly investigating the relationship between oral health status and cognitive impairment and decline on a global scale. Particularly, chronic inflammatory-driven dental disorders suggest that gum inflammation (periodontal disease) is a risk factor for dementia (16). Although numerous studies have found a comorbid association between Alzheimer disease, periodontal disease, and tooth loss (17–20), a 2024 report by the *Lancet* Commission did not find consistent and high-quality evidence to list oral health status as a risk factor for dementia (9). Growing evidence suggests that addressing modifiable risk factors can reduce the risk of developing dementia. These modifiable risk factors include less education, head injury, physical inactivity, smoking, excessive alcohol consumption, hypertension, obesity, diabetes, hearing impairment, depression, infrequent social contact, air pollution, vision impairment, and high cholesterol (9).

The aim of our study was to investigate the relationship between SCD and oral health status and use of dental services, using population-based data. To our knowledge, ours is the first study to jointly examine the relationship between categories of number of teeth lost, use of dental services, and the prevalence of SCD among middle-aged and older adults. By integrating both oral health status and dental care use and adjusting for a broad range of dementia risk factors adopted from the 2024 report by the *Lancet* Commission (9), we aim to provide new epidemiologic insights that extend beyond prior studies focused solely on diagnosed cognitive impairment. We hypothesized that middle-aged and older adults with poorer oral health and lower dental services use would have a higher prevalence of SCD.

## Methods

### Study sample

**Figure 1:**
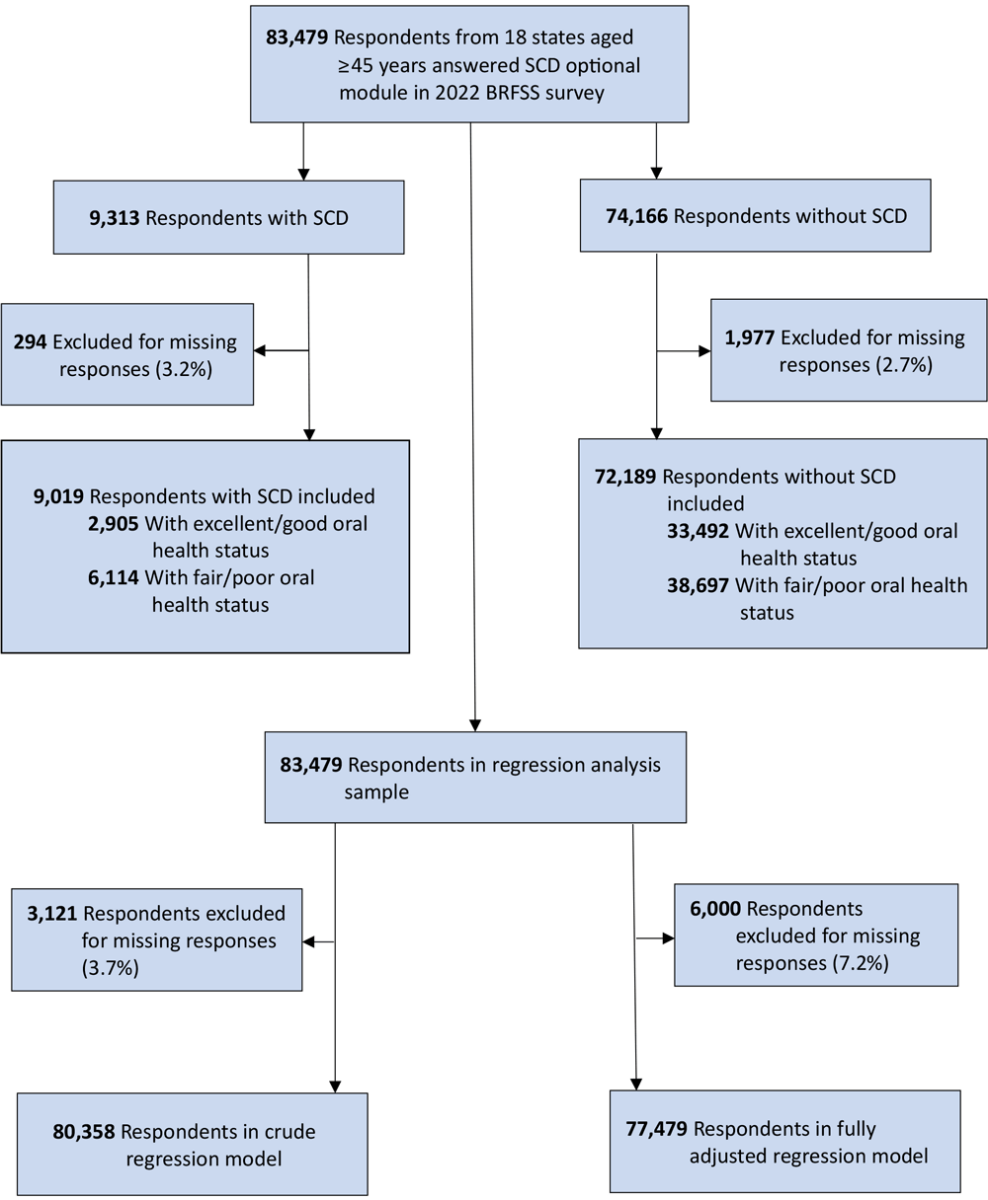
Flowchart of the study sample derivation for analysis of the association between oral health, dental service use, and subjective cognitive decline, 2022 Behavioral Risk Factor Surveillance System (BRFSS) (21). Abbreviation: SCD, subjective cognitive decline.

Our retrospective analysis used de-identified secondary data from the 2022 Behavioral Risk Factor Surveillance System (BRFSS) surveys, available as public use files on the CDC website (21). The BRFSS is a survey of community-dwelling adults aged 18 and older selected through random-digit dialing of landline and cellular phones; details about sampling and data collection procedures are available on the BRFSS website (21). All states administer core questions related to health behaviors, chronic health issues, and prevention services, including oral health, and may choose to include optional modules on topics such as cognitive decline. We included all participants from 18 states aged 45 years and older who responded to an optional module about SCD (N = 83,479) (Table 1). Participants with missing data on exposures, outcome, or covariates were excluded in relevant analyses. A subset of individuals who reported SCD (n = 9,313) was used for descriptive and bivariate analyses (Figure 1).

**Table 1:** Unweighted Number of Respondents Aged 45 Years or Older With and Without Subjective Cognitive Decline, by State, Behavioral Risk Factor Surveillance System, 2022

State	No SCD	SCD	Total
Arizona	2,323	483	2,806
California	2,192	291	2,483
Connecticut	2,357	312	2,669
Florida	6,846	993	7,839
Idaho	2,905	363	3,268
Indiana	5,481	667	6,148
Iowa	2,403	283	2,686
Maine	10,261	1,138	11,399
Michigan	1,838	219	2,057
Nevada	1,548	233	1,781
Ohio	2,784	363	3,147
Oregon	2,799	370	3,169
Rhode Island	3,105	408	3,513
South Carolina	5,576	707	6,283
Utah	4,570	585	5,155
Vermont	5,129	568	5,697
Virginia	5,758	644	6,402
Wisconsin	6,291	686	6,977
Total	74,166	9,313	83,479

Abbreviation: SCD, subjective cognitive decline.

### Measures

**Subjective cognitive decline**. Our primary outcome of interest was subjective cognitive decline. Respondents were asked if they had “experience[d] confusion or memory loss that is happening more often or is getting worse” in the past 12 months. If a respondent answered affirmatively, they were classified as having SCD, and a series of 5 additional questions were asked. These questions were about their daily life, including their level of difficulty with day-to-day activities, need for assistance, availability of help, the impact of SCD on social activities, and whether they had consulted a clinician about their confusion or memory loss. To construct the outcome variable, we categorized respondents as with SCD versus without SCD (22).

### Oral health variables

**Oral health status (number of permanent teeth removed)**. The BRFSS survey asked the following question to ascertain the respondent's loss of teeth due to dental decay or periodontal diseases: “How many of your permanent teeth have been removed because of tooth decay or gum disease? Include teeth lost to infection, but do not include teeth lost for other reasons, such as injury or orthodontics.” Answers were categorized as no missing teeth, 1 to 5 teeth removed, 6 or more but not all teeth removed, all teeth removed (23). Tooth loss due to decay or gum disease has been widely used as a quantitative marker of cumulative oral health burden in population-based research (23,24). Based on established practices, we created a variable to classify those with 1 or more teeth lost due to decay or periodontal disease as having fair or poor oral health and those with no tooth loss as having excellent or good oral health, consistent with methods used in prior studies.

**Use of dental services**. The BRFSS questionnaire assessed dental service use with this question: “Including orthodontists, oral surgeons, and all other dental specialists, as well as dental hygienists, how long has it been since you last visited a dentist or dental clinic for any reason?” We used the American Dental Association guidelines to classify responses as appropriate service use if they reported a visit within 12 months versus inadequate service use if their last visit was over 12 months ago or never (25).

**Covariates.** Based on literature, we adjusted for a minimal sufficient set of potential confounders to estimate the total effect of the number of teeth removed and use of dental services on SCD. Confounders were sociodemographic factors: age, sex, marital status, race/ethnicity, education level, and employment status. General health status was included. We also added the number of chronic conditions based on whether a health care professional ever told the participants they had cardiovascular disease, arthritis, cancer, asthma, chronic obstructive pulmonary disease, or kidney disease. Finally, we defined several dementia risk factors from the 2024 *Lancet* report (9): Physical inactivity as no physical activities or exercises during the past month; binge alcohol drinking as having 5 or more drinks on at least one occasion for men or 4 or more drinks for women; smoker, as currently smoking cigarettes every day, some days, or not at all, if the respondent had smoked at least 100 cigarettes in their entire life; obesity, as a body mass index (BMI) of 30 or more; living alone as being the only adult in the household and no children were present; hearing impairment as a yes response to the question, “Are you deaf or do you have serious difficulty hearing?”; vision impairment as a yes response to “Are you blind or do you have serious difficulty seeing, even when wearing glasses?”; diabetes if a clinician had ever told the respondent they had diabetes mellitus, other than during pregnancy; and depression as having “ever been diagnosed with a depressive disorder including depression, major depression, dysthymia, or minor depression.”

### Statistical analyses

We calculated weighted percentages for all variables in descriptive analyses. By using weighted estimates (percentages), we conducted bivariate analysis of SCD and oral health status (excellent/good vs fair/poor). Chi-square tests (*P* < .05) were used to assess the relationship between SCD, oral health, sociodemographic factors, chronic diseases, and dementia risk factors. We used a generalized linear model with binomial family and log link, to calculate prevalence ratios (PR) (26). We tested for effect modification by age group (45–64 and ≥65 y) by using multiplicative interaction terms between each primary exposure and age group. We found a significant interaction and therefore present results stratified by age group. We ran a model adjusted for sociodemographic factors, chronic diseases, and dementia risk factors. We used Stata 18.0 (StataCorp). All analyses, including the generalized linear model, used svy statements and incorporated the primary sampling unit, stratum, and weight information to account for BRFSS's complex sample design. Sample sizes are provided as unweighted counts. We considered differences significant at *P* < .05.

## Results

Of the 83,479 respondents who completed the BRFSS’s SCD module, 9,313 reported experiencing subjective cognitive decline. The prevalence of SCD was higher among people aged 45 to 64 years (12.2%) than people aged 65 or older (10.1%) (*P* < .001 for both). About half of respondents with SCD were female (52.1%), non-Hispanic White (67.1%), married (51.8%), and in excellent, very good, or good general health (53.7%) Nearly half of respondents with SCD had 2 or more chronic conditions (49.7%), and most had 2 or more dementia risk factors (79.2%) (Table 2).

**Table 2:** Sociodemographic Characteristics, Chronic Conditions^a^, and Dementia Risk Factors^b^ Among Participants Aged 45 Years or Older With Subjective Cognitive Decline (N = 9,019), by Oral Health Status, Behavioral Risk Factor Surveillance System, 2022

Characteristic	Fair/poor oral health, with SCD, n (weighted%) (n = 6,114)	Excellent/good oral health with SCD, n (weighted%) (n = 2,905)	*P *value^d^
**Age, y^c^**			
45–64	2,472 (49.1)	1,445 (58.7)	<.001
≥65	3,642 (50.9)	1,460 (41.3)	
**Sex^c^**			
Female	3,307 (51.4)	1,632 (53.9)	.40
Male	2,807 (48.6)	1,273 (46.1)	
**Race or ethnicity^c^**			
Non-Hispanic Asian	41 (4.3)	23 (4.7)	.84
Non-Hispanic Black	422 (8.9)	108 (5.4)	.01
Hispanic	305 (12.8)	155 (14.1)	.64
Non-Hispanic White	4,905 (66.0)	2,460 (70.9)	.16
Other or multiple non-Hispanic races	226 (8.1)	61 (4.9)	.07
**Marital status^c^**			
Married	2,674 (48.0)	1,699 (60.7)	<.001
Not married	3,402 (52.1)	1,186 (39.3)	
**Education^c^**			
Less than high school graduate	602 (18.8)	91 (7.0)	<.001
High school graduate	1,856 (28.9)	566 (22.7)	.02
Some college	1,944 (33.5)	818 (35.3)	.51
College graduate	1,688 (18.6)	1,418 (34.9)	<.001
**Employment**			
Employed	1,210 (22.8)	1,009 (39.2)	<.001
Unemployed	294 (7.7)	102 (4.8)	.18
Retired	3,040 (40.4)	1,317 (40.0)	.88
Not working	1,529 (28.7)	458 (15.8)	<.001
Fair or poor general health	3,049 (51.7)	959 (34.3)	<.001
**Number of chronic conditions^c^**			
None	968 (16.8)	752 (26.8)	<.001
1	1,910 (33.5)	1,074 (38.5)	
≥2	3,236 (49.7)	1,079 (34.6)	
**Number of dementia risk factors^c^**			
None	339 (5.7)	392 (14.6)	<.001
1	927 (15.0)	672 (25.5)	
≥2	4,848 (79.2)	1,841 (59.9)	

Abbreviation: SCD, subjective cognitive decline.

a Cardiovascular diseases, arthritis, cancer, asthma, chronic obstructive pulmonary disease, or kidney disease.

b Physical inactivity, binge alcohol drinking, smoking, obesity, living alone, hearing impairment, vision impairment, depression, diabetes.

c Variable was included in the fully adjusted regression model.

d Calculated by χ^2^. Differences between participants with subjective cognitive decline with excellent/good oral health status or fair/poor oral health status significant at *P* < .05.

### Subjective cognitive decline, oral health status, and dental services use

**Figure 2:**
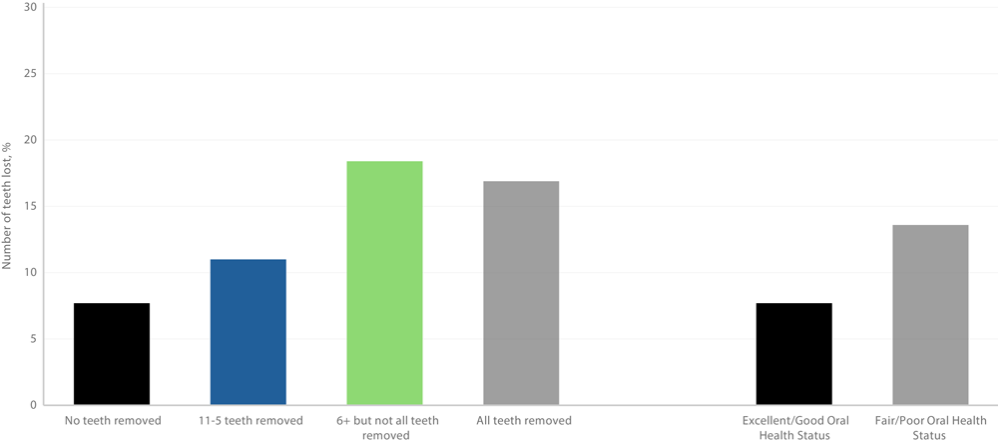
Weighted prevalence of subjective cognitive decline among adults aged 45 years by number of teeth removed and oral health status (N = 83,479), Behavioral Risk Factor Surveillance System 2022 (21).

Of 83,479 respondents to the SCD module, 2,278 were excluded because of missing data on oral health variables. Respondents with SCD had 6 or more teeth removed, but not all teeth (22.4% vs 12.1%), and had a lower prevalence of visiting a dentist in the past year (57.7% vs 69.3%) compared with those without SCD (Table 3). Crude estimates showed higher SCD prevalence among respondents with fair to poor oral health status (13.6%) than among people with excellent to good oral health status (7.7%) (Figure 2). SCD prevalence increased with more tooth loss: 11.0% for 1 to 5 teeth lost, 18.4% for 6 or more but not all, and 16.9% for all teeth lost. Among respondents who had not used dental services within the last year, 14.4% had SCD compared with 9.2% of those who used dental services within the last year.

**Table 3:** Characteristics of Participants Aged 45 Years or Older (N = 83,479) With and Without Subjective Cognitive Decline (SCD) and Their Oral Health Status, Number of Teeth Removed, and Use of Dental Services, Behavioral Risk Factor Surveillance System, 2022

Characteristic	Total number without SCD (n = 74,166)^a^	Total number with SCD (n = 9,313)^a^	*P *value^b^
**Oral health status**			
Good	33,492 (47.2)	2,905 (32.1)	<.001
Fair/poor	38,114 (52.7)	6,114 (67.8)	
**Number of teeth removed**			
None	33,492 (47.2)	2,905 (32.1)	<.001
1–5	24,107 (33.8)	3,049 (34.2)	
≥6	9,149 (12.1)	1,882 (22.4)	
All	5,441 (6.7)	1,183 (11.2)	
**Used dental services in the past year**			
Yes	52,922 (69.3)	5,578 (57.7)	<.001
No	5,578 (30.6)	3,597 (42.4)	

a Values are n (weighted %).

b Calculated by χ^2^. Differences between participant with and without subjective decline with good oral health status or bad/poor oral health status was at *P* < .05.

**Table:** 

Prevalence, subjective cognitive decline	Percentage
No teeth removed	7.7
1-5 teeth removed	11.0
>6 but not all teeth removed	18.4
All teeth removed	16.9
Excellent/good oral health status	7.7
Fair/poor oral health status	13.6

In regression models, 3.7% of respondents were excluded from the crude model and 7.2% from the fully adjusted model because of missing data. In both crude and fully adjusted models, all tooth-loss categories were significantly associated with higher prevalence of reporting SCD compared with people with no tooth loss (Table 4). In the age-stratified, fully adjusted model, middle-aged adults with 1 to 5 teeth removed had a 25% higher prevalence of SCD (PR = 1.25, 95% CI, 1.04–1.50, *P* = .02). Estimated SCD prevalence was higher for those with 6 or more teeth removed (PR = 1.91, 95% CI,1.50–2.44, *P* < .001). For complete tooth loss, SCD prevalence was lower than for those with 6 teeth lost (PR = 1.37; 95% CI, 1.07–1.76, *P* = .013). Within this age group, people who had visited a dentist twice per year had a significantly lower SCD prevalence than those who had not (PR = 0.77; 95% CI, 0.65–0.90, *P* = .002). Among older adults, having 1 to 5 teeth removed (PR = 1.19; 95% CI, 1.00–1.41, *P* = .04) and 6 or more teeth removed (PR = 1.35; 95% CI, 1.07–1.69, *P* = .01) was also associated with a higher prevalence of SCD. However, the associations for complete tooth loss and last dental visit were not significant (Table 4).

**Table 4:** Prevalence Ratios for the Association Between Subjective Cognitive Decline, Oral Health Status, and Use of Dental Services Among Adults Aged 45 Years or Older (N = 83,479), Behavioral Risk Factor Surveillance System, 2022

Variable	Crude model, n = 80,358				**Fully-adjusted model^b^, n = 77,479**			
	Prevalence Ratio (95% CI) Age 45–64, n = 37,318	*P *value^a^	Prevalence Ratio (95% CI) Age ≥65, n = 43,040	*P *value^a^	Prevalence Ratio (95% CI) Age 45–64, n = 35,986	*P *value^a^	Prevalence Ratio (95% CI) Age ≥65, n = 41,493	*P *value^a^
**Number of teeth lost (reference: none) **								
1–5 teeth removed	1.43 (1.19–1.71)	<.001	1.28 (1.07–1.53)	.005	1.25 (1.04–1.50)	.02	1.19 (1.00–1.41)	.04
6 or more teeth removed, but not all teeth	2.87 (2.30–3.59)	<.001	1.61 (1.30–2.00)	<.001	1.91 (1.50–2.44)	<.001	1.35 (1.07–1.69)	.01
All teeth removed	2.46 (1.95–3.11)	<.001	1.45 (1.15–1.82)	.001	1.37 (1.07–1.76)	.01	1.17 (0.93–1.49)	.17
**Last dental visit (reference: >1 year ago or never)**	0.65 (0.55–0.76)	<.001	0.81 (0.68–0.97)	.029	0.77 (0.65–0.90)	.002	0.93 (0.78–1.11)	.45

a Calculated by generalized linear model with binomial family and log links. Significant at *P* < .05.

b Fully adjusted regression model includes race or ethnicity, marital status, sex, level of education, number of dementia risk factors, and number of chronic conditions.

## Discussion

This cross-sectional study suggests a significant association between tooth loss and use of dental services and SCD, even after accounting for sociodemographic factors, chronic conditions, and dementia risk factors. People with more missing teeth generally had a higher prevalence of SCD, while regular dental visits were associated with a lower model-estimated prevalence. Our findings align with previous case-control, cohort, and longitudinal studies that reported associations between substantial tooth loss (losing 6 or more teeth) and cognitive decline (3,20,22,23). However, our study adds new perspectives after age-stratification by examining multiple tooth loss categories rather than a single threshold, and by incorporating use of dental services with oral health status within the same regression models. It offers a more comprehensive view of how both oral health status and use of dental services relate to SCD.

We found that SCD prevalence generally increased when the number of teeth lost increased. However, this trend did not occur in the group with all teeth removed. It is unclear why the prevalence of SCD did not increase in a linear way with increasing tooth loss as we expected. We speculate that this could be because a person who has lost all their teeth could receive prosthodontic treatment, such as dental implants or dentures. A dose–response meta-analysis study by Qi et al provides a possible explanation: they found that denture use could mitigate the risk of dementia or cognitive impairment (12).

A US study established a link between masticatory function (chewing) and dementia, suggesting that people without teeth who wore dentures improved their masticatory function, potentially reducing their risk of dementia compared with those without dentures (27). A Japanese study found that people with fewer teeth and no dentures had a dementia risk nearly double that of people who had dentures to replace lost teeth and attributed the increased risk to general health status and health behaviors (29). Thus, masticatory function appears important, warranting further research. 

In an evidence summary, Daly et al reported that people with dementia often experience worse oral health, with a strong and consistent link between poor oral hygiene and cognitive decline (29). In addition, we found a significant association between use of dental services and SCD among our middle-aged group. Even after adjusting for confounders, it appears that visiting the dentist as recommended could be a protective factor against SCD. Maintaining good oral health could potentially contribute to the preservation of cognitive function. This finding aligns with the findings of a study conducted by Scherer and Scherer, which showed a correlation between regular dental care and a decreased incidence of SCD in people aged 45 years or older (20). However, it remains unclear whether the reduction in periodontal disease severity is the underlying cause of this association. Our finding agrees with 1 study, which found no significant association between dental visits and cognitive decline among people aged 64 years or older (30). Additionally, another study reported that no regular dental visit and poor oral hygiene were predictors for incidents of dementia (28). Interestingly, in a recently published systematic review and meta-analysis of oral hygiene behaviors, regular teeth brushing, twice a day, was linked to a reduction of dementia risk (31).

Several explanations are possible for the observed association between tooth loss and diminished cognitive function. In line with the idea that dentures can be helpful, some studies suggested that difficulty with proper chewing after losing teeth may cause changes in the structure or function of certain brain areas involved in cognition (32,33). A 5-year prospective study by Okamoto et al found a significant association between a higher number of teeth lost and the development of mild memory impairment, which was attributed to reduced masticatory function and early cognitive decline due to reduced masticatory stimulation of brain regions (34). Another possible explanation is that tooth loss and suboptimal masticatory function contribute to nutritional deficiencies, leading to the loss of key nutrients for brain health (12,34). For instance, vitamin D has been shown to reduce the expression of cytokines linked to periodontal disease and dementia, such as interleukin-6, interleukin-8, and tumor necrosis factor-α (35,36). Lastly, inflammation in the mouth and harmful oral bacteria such as *Porphyromonas gingivalis* may affect neuroinflammatory processes and the production of β-amyloid (37). Furthermore, inflammatory cytokines, such as C-reactive protein, tumor necrosis factor-α, immunoglobulin G, interleukin-1β, and interleukin-6, are released in response to periodontitis. These cytokines contribute to the inflammatory process, which is known as an etiological factor for tooth loss (12,38).

Although most of the studies mentioned here looked at people aged 65 years or older except for Scherer and Scherer (20), study participants may have already had dementia or cognitive impairment. These conditions may increase the risk of tooth loss because of poor oral hygiene, especially without self-care or assisted oral care practices or regular dental visits (39). Our study is unique in that it looked at middle-aged and older adults with SCD and found that oral health status and dental services use had a significant impact on SCD; however, given the cross-sectional nature of BRFSS, the observed associations in our study cannot be interpreted as causal or directional. While we found significant relationships, our study’s design did not allow us to determine which condition preceded the other or resulted from it. As such, the reported PRs should be interpreted as noncausal associations within the sample rather than measures of risks.

Our cross-sectional study had several limitations. First, self-reported data for exposure variables and outcome could lead to bias and misclassification. Participants may struggle to accurately report earlier tooth extractions, and those with SCD symptoms may not report them because of memory problems or stigma, which could underestimate SCD prevalence (2), thereby biasing the observed associations. BRFSS’s voluntary nature could introduce selection bias, causing us to underestimate associations if cognitively impaired people participated less. Second, BRFSS is a telephone-based survey that uses random-digit dialing of landlines and cellular telephones for noninstitutional households. The survey includes a representative sample of the US population and generalizable results, but it excludes adults living in institutional settings, such as nursing homes, who may have higher rates of poor oral health and cognitive impairment. This exclusion may limit the applicability of our results to those vulnerable populations. Additionally, our analysis sampled adults aged 45 years and older who completed the cognitive decline module. Missing data were relatively uncommon in this sample, but we acknowledge that respondents with incomplete data may differ systematically from those included in the analysis. This could introduce selection bias, the direction of which we were unable to evaluate directly. We hypothesize that people with SCD would be more likely to under-report tooth loss than people without SCD because of memory problems, which would make the association appear stronger in the lower tooth-loss categories and weaker in the higher tooth-loss categories. Third, BRFSS does not report denture use among participants who have lost all their teeth (22). Fourth, we could not incorporate all the dementia risk factors listed by both the 2023 and 2024 *Lancet* Commission reports (9), such as hypertension and high cholesterol, because the optional modules associated with these variables are administered every other year, and others are not included in BRFSS. The absence of these variables may cause residual confounding, which may weaken or strengthen outcome–exposure associations. Although important, we could not account for shared genetics that influence inflammatory pathways or neurodegenerative processes and may have influenced our observation. Fifth, although time since last dental visit provides a measure of use of dental services, it does not capture the quality, comprehensiveness, or the type of dental services received. Lastly, the cross-sectional nature of the data makes it impossible to establish cause-and-effect relationships. However, using a large amount of BRFSS data allows for a diverse demographic sample and a large, representative sample size.

In conclusion, our study reinforces previous evidence that oral health status, particularly tooth loss, may be linked to increased risk of dementia. Both periodontal disease and dental decay contribute to tooth loss — periodontal disease through its inflammatory-driven effect on supporting structure, and dental decay through the progressive breakdown of tooth structure. We found that visiting a dentist at least annually is associated with a lower SCD prevalence in our study’s middle-aged group, likely because of preventive care that helps maintain oral health. Unexpectedly, middle-aged people who had all their teeth removed had a lower prevalence of SCD compared with those who lost 6 or more teeth but not all, possibly due to receiving prosthodontic treatment that supports masticatory function. Among older adults, no significant association was observed for those who had all their teeth removed. These observations suggest that dental rehabilitation may help lower cognitive risks. Therefore, we should interpret our findings cautiously because of our study’s cross-sectional design. Longitudinal studies are needed to assess and clarify temporal relationships and potential mechanisms linking oral health and cognitive decline. Nonetheless, our findings suggest potential clinical and public health implications that could guide oral health promotion efforts for adults at risk of SCD. Health care providers — including dentists, primary care clinicians, and public health professionals — can play an important role in educating patients, family members, and caregivers on the importance of maintaining oral health as part of overall health. The implementation of community-based dental health promotion programs and interventions has the potential to benefit a range of health issues, including cognitive health.
